# Polypyrrole–Nickel Hydroxide Hybrid Nanowires as Future Materials for Energy Storage

**DOI:** 10.3390/nano9020307

**Published:** 2019-02-24

**Authors:** Agnieszka Brzózka, Krzysztof Fic, Joanna Bogusz, Anna M. Brudzisz, Mateusz M. Marzec, Marta Gajewska, Grzegorz D. Sulka

**Affiliations:** 1 Jagiellonian University in Krakow, Department of Physical Chemistry and Electrochemistry, Gronostajowa 2, 30-387 Krakow, Poland; asia_bogusz_@onet.eu (J.B.); anna.brudzisz@doctoral.uj.edu.pl (A.M.B.); 2 Institute of Chemistry and Technical Electrochemistry, Poznan University of Technology, Berdychowo 4, 60-965 Poznan, Poland; krzysztof.fic@put.poznan.pl; 3 AGH University of Science and Technology, Academic Centre for Materials and Nanotechnology, A. Mickiewicza 30, 30-059 Krakow, Poland; marzecm@agh.edu.pl (M.M.M.); marta.gajewska@agh.edu.pl (M.G.)

**Keywords:** polymer-inorganic hybrid structure, supercapacitors, potential pulse electrodeposition

## Abstract

Hybrid materials play an essential role in the development of the energy storage technologies since a multi-constituent system merges the properties of the individual components. Apart from new features and enhanced performance, such an approach quite often allows the drawbacks of single components to be diminished or reduced entirely. The goal of this paper was to prepare and characterize polymer-metal hydroxide (polypyrrole-nickel hydroxide, PPy-Ni(OH)_2_) nanowire arrays demonstrating good electrochemical performance. Nanowires were fabricated by potential pulse electrodeposition of pyrrole and nickel hydroxide into nanoporous anodic alumina oxide (AAO) template. The structural features of as-obtained PPy-Ni(OH)_2_ hybrid nanowires were characterized using FE-SEM and TEM analysis. Their chemical composition was confirmed by energy-dispersive x-ray spectroscopy (EDS). The presence of nickel hydroxide in the synthesized PPy-Ni(OH)_2_ nanowire array was investigated by X-ray photoelectron spectroscopy (XPS). Both FE-SEM and TEM analyses confirmed that the obtained nanowires were composed of a polymer matrix with nanoparticles dispersed within. EDS and XPS techniques confirmed the presence of PPy-Ni(OH)_2_ in the nanowire array obtained. Optimal working potential range (i.e., available potential window), charge propagation, and cyclic stability of the electrodes were determined with cyclic voltammetry (CV) at various scan rates. Interestingly, the electrochemical stability window for the aqueous electrolyte at PPy-Ni(OH)_2_ nanowire array electrode was remarkably wider (ca. 2 times) in comparison with the non-modified PPy electrode. The capacitance values, calculated from cyclic voltammetry performed at 20 mV s^−1^, were 25 F cm^−2^ for PPy and 75 F cm^−2^ for PPy-Ni(OH)_2_ array electrodes. The cyclic stability of the PPy nanowire array electrode up to 100 cycles showed a capacitance fade of about 13%.

## 1. Introduction

The research on the new energy-related materials (NERM) appears to be very significant now that the energy demand and consumption is rapidly increasing. It is widely accepted that conventional materials, based on the various oxides, conducting polymers, and hybrid materials, need to be re-designed at the nano-scale in order to fully exploit their electrochemical potential [1]. Quite often, such manipulation allows the negative impact of the diffusion to be reduced and rate capability to be improved [2,3]. Nanostructured materials as “building blocks” of electrochemical capacitors (ECs), batteries, and low-temperature fuel cells have recently attracted much interest [2]. While low-temperature fuel cells and batteries (like Li-ion or Ni-MH) are characterized by low power (up to 2 kW kg^−1^) and high energy density (up to 1 kWh kg^−1^), electrochemical capacitors may deliver a power density of 100 kW kg^−1^ at an energy density usually up to 15 Wh kg^–1^ [4]. On the one hand, it is said that electrochemical capacitors cannot replace batteries or fuel cells. On the other hand, their application niche is definitely different [5,6,7] and such a claim seems to be pointless. Notwithstanding, ECs might greatly complement other energy storage devices like batteries, protecting them against current fluctuations (at high power demand) and prolonging the system lifetime.

Electrochemical capacitor is an energy storage device that can be used in a vast range of applications, including portable electronics, large-scale power generators, and hybrid or electric vehicles [7]. Fast charging (within seconds), reliability, high stability over long-term cycling (up to a few thousands of cycles), and high power density (from 0.1 to 100 kW kg^–1^) [8] indicate that ECs can find a number of applications where a conventional, low-power source (like battery) cannot be used. Additionally, ECs could be used for energy recovery during repetitive processes, such as braking in vehicles (essentially in public transport) or load descending in lifts or cranes, due to their fast charging rate [8].

The mechanism of energy storage in electrochemical capacitors differentiates them for two main categories: electric double-layer capacitors (EDLCs) and supercapacitors [9]. However, one should be aware that the term “supercapacitor” is misleading and not recommended by the community since it is usually confused with double-layer-based devices. EDLCs store the electric energy in the electric double-layer, formed at the electrode/electrolyte interface. Thus, the energy is stored purely on the electrostatic manner. Supercapacitors store the energy by two mechanisms: in an electric double-layer (electrostatic interactions) and by redox-based phenomena, providing so-called pseudocapacitance (PC) [4]. However, it has to be mentioned that the term “pseudocapacitance” is recently quite often overused and confused with typical faradaic storage [10,11,12].

To date, various metal oxides and conducting polymers have been extensively studied as pseudocapacitive materials [13]. Conducting polymers, e.g., poly(3,4-ethylenedioxythiophene) (PEDOT), polyaniline (PANI), and polypyrrole (PPy), as organic polymers with electronic properties typical of semiconductors, can be used as future materials for data storage, photovoltaic cells, and supercapacitors [14,15,16]. Furthermore, polymer-based materials can also be used for template-assisted electrosynthesis of hybrid metal-polymer nanowires [17] and deposition or dispersion of the particles in the polymer matrix [18,19]. Metal oxides, in the form of thin films or nanostructures, can also be used as the electrode material for fuel cells, lithium-ion batteries, and electrochemical supercapacitors [20]. Considerable scientific attention has been directed towards the use of NiO-based electrodes in high-performance ECs, because of the low-cost and facile synthesis with a diversity of approaches [14,21,22,23,24,25]. Some reports suggested that the nickel hydroxide can also be successfully used as the material for electrochemical supercapacitors [26,27,28,29,30,31,32,33,34,35,36,37,38,39]. However, its electrochemical performance suggests somewhat hybrid energy storage mechanisms in the devices exploiting this material [20].

An increasing number of reports about the use of hybrid/composite materials in electrochemical capacitors, e.g., polymer with carbon/graphene [40,41,42,43], tin oxide [44,45,46], ruthenium oxide [47,48], manganese oxide [49,50], or nickel hydroxide [51,52,53,54,55,56,57], can be found in the literature. It seems that the application of hybrid material in energy storage devices improves their performance because these multicomponent systems combine the properties of their components. This can result in the elimination of the disadvantages of individual components.

Since the capacitance of electrochemical capacitors is proportional to the electrochemically accessible surface area at the electrode/electrolyte interface, the active surface of the electrode material is of great importance. Furthermore, one-dimensional nano-objects can diminish the impact of the diffusion resistance of an electrolyte species during the charge–discharge process. Hence, the hybrid polymer-transition metal hydroxide nanowire arrays seem to be an excellent material for the fabrication of electrodes for supercapacitors. To our knowledge, no previous reports on the preparation of polymeric nanowires modified with nanoparticles of Ni(OH)_2_ in electrochemical capacitors have been published. The obvious advantages of such material are the low cost and simple electrode preparation when the nanoporous anodic aluminum oxide (AAO) is used as the template.

The purpose of this study was to obtain and characterize a new hybrid material based on PPy-Ni(OH)_2_ nanowire array. It was expected that this material would demonstrate a high capacitance. The hybrid polymeric-metal hydroxide nanowires were fabricated by potential pulse electrodeposition of pyrrole and nickel hydroxide into the nanoporous anodic alumina oxide template. A comprehensive characterization of the synthesized hybrid nanowire arrays was done, and the obtained results indicated that the material synthesized this way shows promising electrochemical behavior.

## 2. Materials and Methods

### 2.1. Fabrication of AAO Templates

Through-hole AAO template with hexagonally arranged cylindrical nanopores was prepared using two-step anodization of Al foil, followed by an electrochemical detachment process [58,59]. A high purity aluminum foil (99.999%, GoodFellow, Huntingdon, United Kingdom) was degreased in acetone (Avantor Performance Materials Poland S.A., Gliwice, Poland, p.a.) and ethanol (Avantor Performance Materials Poland S.A., Gliwice, Poland, 96%), and then electrochemically polished in an unstirred mixture of perchloric acid (Avantor Performance Materials Poland S.A., Gliwice, Poland, 70%) and ethanol (1:3 vol.). The process of electropolishing was conducted in a conventional two-electrode cell with an Al plate as an anode and Pt grid as a cathode. The temperature during the process was maintained at 0 °C, and a constant voltage of 20 V for 2 min was applied in order to obtain a mirror finish aluminum surface. Two-step anodization of aluminum was performed in a two-electrode electrochemical cell in a 0.3 M H_2_C_2_O_4_ (Avantor Performance Materials Poland S.A., Gliwice, Poland, p.a.) solution at 20 °C and at the constant voltage of 45 V. The duration of the first and second anodization steps was 1 h and 4 h, respectively. Typically, a disordered oxide layer that was formed in the first step of anodization was removed in a mixture of 6 wt% H_3_PO_4_ (Avantor Performance Materials Poland S.A., Gliwice, Poland, 85%)and 1.8 wt% H_2_CrO_4_ (Avantor Performance Materials Poland S.A., Gliwice, Poland, p.a.) at 60 °C for 1 h. This process allows for the removal of the resulting aluminum oxide film and, consequently, reveals a concave array on the anodized Al surface. After the two-step anodization of aluminum, the through-hole AAO membrane was obtained by the voltage detachment method [59]. The process of voltage detachment was conducted in a mixture of perchloric acid and ethanol in the volume ratio of 1:1. During the voltage detachment, the temperature of 0 °C was maintained in the electrochemical cell and three pulses of 60 V for 3 s each were applied. After the mechanical separation of the membrane from the Al substrate, the samples were thoroughly rinsed with distilled water and finally air-dried.

### 2.2. Preparation of PPy-Ni(OH)_2_ Nanowire Array Electrodes

**Figure 1:**
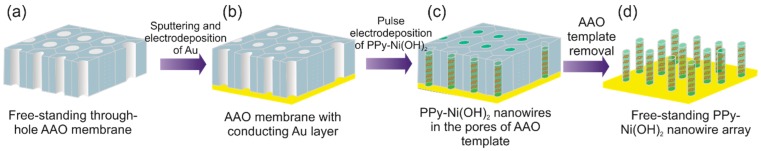
Schematic representation of the synthesis of free-standing PPy-Ni(OH)_2_ nanowire array electrodes.

The PPy-Ni(OH)_2_ nanowire array electrodes were synthesized according to the procedure presented in Figure 1.

**Figure 2:**
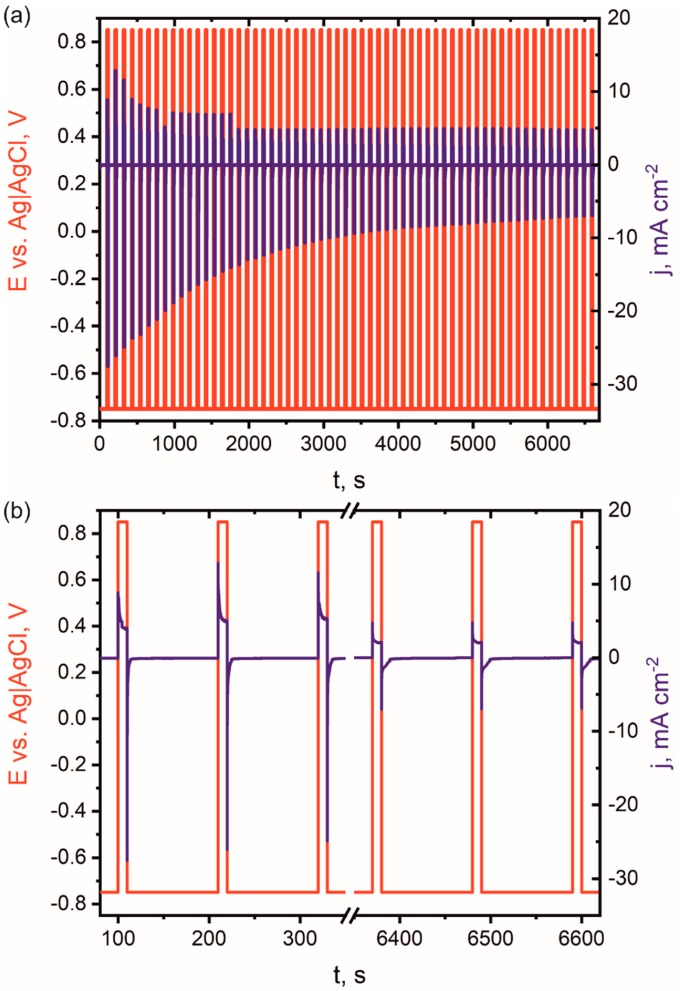
Time profiles of the applied potential and resulting current density for the whole electrodeposition (**a**) and 3 first and last pulses (**b**).

Free-standing AAO membrane with a thickness of about 40 µm was obtained as a result of the anodization and voltage detachment procedure (Figure 1a). To enable electrochemical deposition, one side of the non-conductive AAO membrane was covered with a thin conductive Au layer by using a vacuum sputter coater (Emitech K575X, Laughton, United Kingdom). A thin layer of Au was then thickened by electrochemical deposition of gold from a commercially available gold plating solution (Umicore, Hanau, Germany, Auruna® 5000, Au content 7 g dm^−3^), by applying a constant current density of 1 mA cm^−2^ (Figure 1b). After that, the AAO/Au template (gold served later as a current collector) was fixed in a Teflon® coated electrode, which was placed in the three-electrode electrochemical cell. The working surface area of the AAO membrane was made constant by using the O-ring with the inner diameter of 3 mm. The synthesis of polypyrrole-Ni(OH)_2_ hybrid nanowire array (Figure 1c) was performed in a three-electrode configuration using the Teflon® coated Au/AAO template as a working electrode, Ag|AgCl as a reference electrode, and a Ti grid as a counter electrode. The electrodeposition of hybrid nanowires was carried out at room temperature using a single Watts-type nickel bath (NiSO_4_∙6H_2_O (Sigma Aldrich, Saint Louis, MO, USA, 98%), NiCl_2_∙6H_2_O (Sigma Aldrich, Saint Louis, MO, USA, 97%), H_3_BO_3_ (Avantor Performance Materials Poland S.A., Gliwice, Poland, 99.5%)) [60] containing 0.15 M pyrrole monomer (Sigma Aldrich, Saint Louis, MO, USA, 98%) with 0.10 M LiClO_4_ (Sigma Aldrich, Saint Louis, MO, USA, 95%), and 0.10 M Na_2_CO_3_ (Sigma Aldrich, Saint Louis, MO, USA, 99.5%). The pH value of the solution was adjusted to 4–5 by hydrochloric acid (Avantor Performance Materials Poland S.A., Gliwice, Poland, 35–38%) or sulfuric acid (Sigma Aldrich, Saint Louis, MO, USA, 95–98%) in order to prevent the precipitation of poorly soluble nickel carbonate [61]. After the potential pulse electrodeposition, a free-standing hybrid PPy-Ni(OH)_2_ nanowire array was obtained by chemical etching of the AAO template in a 1 M NaOH solution (Figure 1d). A pulse waveform applied during deposition is depicted in Figure 2.

### 2.3. Samples Characterization

The morphology characterization of the fabricated hybrid PPy-Ni(OH)_2_ nanowire array electrode was performed by field emission scanning electron microscopy (FE-SEM Hitachi S-4700, Tokyo, Japan) and transmission electron microscopy (TEM). TEM analyses were performed using an FEI Tecnai TF20 X-TWIN (FEG, Hillsboro, OR, USA) microscope at an accelerating voltage of 200 kV.

The chemical composition of the hybrid PPy-Ni(OH)_2_ nanowire array was examined by energy-dispersive x-ray spectroscopy (EDAX Noran System 7, Tokyo, Japan and EDAX RTEM 0.3 sr, HAADF, Hillsboro, OR, USA) and X-ray photoelectron spectroscopy (XPS). The X-ray photoelectron spectroscopy investigation was performed in a PHI VersaProbeII Scanning XPS system (Ulvac-Phi, Chigasaki, Japan) using monochromatic Al Kα (1486.6 eV) X-rays focused on a 100 µm spot and rastered over an area of 400 × 400 µm^2^. In order to achieve high energy resolution spectra for the Ni 2p_3/2_ region, pass energy in the analyzer was fixed at 23.50 eV and the photoelectron take-off angle was set to 45°. Dual beam charge compensation with 7 eV Ar^+^ ions and 1 eV electrons was applied to maintain a constant sample surface irrespective of the sample conductivity. The collected XPS spectra were charge referenced to the unfunctionalized, saturated carbon (C-C) C 1s peak at 284.8 eV. The working pressure in the analytical chamber was less than 4 × 10^−9^ mbar. Deconvolution of spectra was carried out using PHI MultiPak software (v.9.8.0.19, Chigasaki, Japan) with spectrum background subtracted using the Shirley method.

### 2.4. Study of Electrochemical Properties of PPy-Ni(OH)_2_ Electrodes

The electrochemical characterization of the PPy-Ni(OH)_2_ nanowire array electrode was performed using a multi-channel potentiostat/galvanostat VMP 3 (BioLogic®, Seyssinet-Pariset, France). Cyclic voltammetry (CV) measurements in a three-electrode configuration determined the electrochemical potential window and capacitance vs. scan rate dependence. The PPy-Ni(OH)_2_ hybrid nanowire array was used as a working electrode, while the saturated calomel electrode (SCE) and Pt coil served as a reference and counter electrode, respectively. The CV experiments were conducted in an electrolyte consisting of 1 M LiSO_4_ + 0.19 M 1,4-dihydroxybenzene aqueous solution (pH = 5.5) at room temperature. Additionally, the cyclic stability of a polypyrrole nanowire array electrode was verified by cyclic voltammetry in the electrolyte consisting of 1 M LiSO_4_ + 0.19 M 1,4-dihydroxybenzene aqueous solution (pH = 5.5) at room temperature.

## 3. Results and Discussion

The AAO membrane with a pore diameter of ~60 nm was used as a template for the fabrication of PPy-Ni(OH)_2_ hybrid nanowire array.

The phenomenon of formation of hybrid nanowires was identified by detailed analysis of working electrode potential vs. time and current density vs. time curves (Figure 2).

The hybrid nanowires were electrodeposited from a single bath containing bath components Ni^2+^ ions and pyrrole monomer. Polypyrrole is a π-conjugated polymer and can be easily synthesized by anodic electropolymerization from an aqueous solution [62], while metal (Me) reduction occurs at negative potentials. Although it is possible to obtain hybrid PPy-Me nanowires by cathodic co-deposition [63], at potentials more negative than –0.628 V vs. Ag|AgCl, we decided to synthesize hybrid nanowires by pulse alternating electrodeposition in which cathodic and anodic pulses were used to form Ni(OH)_2_ nanoparticles and PPy nanowires, respectively. When the working electrode potential is set at –0.750 V vs. Ag|AgCl, the modulus of cathodic current density increases immediately to a certain value, i.e., 27.52 mA cm^–2^. After a few seconds, the modulus of cathodic current density decreases due to the formation of Ni(OH)_2_ in a steady-state growth process. The modulus of cathodic current density at the stable growth of Ni(OH)_2_ is ca. 0.06 mA cm^−2^. The equilibrium reduction potential of nickel is –0.225 V [64], but the overpotential of nickel reduction and morphology of final product depends on several factors such as pH, ion concentration, electrode material, and bath additives [64,65]. During the cathodic reduction of nickel ions in an aqueous solution, two main reactions occur, i.e., nickel electroplating (Equation (1)) and hydrogen evolution (Equation (2)) [64].

(1) $$Ni^{2+}+2e^{-}\to Ni$$

(2) $$2H_{2}O+2e^{-}\to 2OH^{-}+H_{2}.$$

These reactions occur at more negative potentials than those applied in this work. Nickel ion electroreduction occurs at the potential, ca. −1.88 V vs. SCE in the Watts-type bath, at the same potential H_2_ evolution, is observed.

As the potential of the working electrode during the experiment was less negative than the potential of nickel electrodeposition, only hydrogen evolution reaction is possible. When the hydrogen evolution reaction is observed, the hydroxide ions are produced, at the same time, near the electrode surface (Equation (2)). The hydroxide ions can react with nickel ions, so a possible product of electroreduction is nickel hydroxide, formed during the following reaction (Equation (3)) [66]:

(3) $$Ni^{2+}+2OH^{-}\to Ni{\left(OH\right)}_{2}.$$

The possibility of Ni(OH)_2_ formation during nickel electrodeposition at less negative potentials, ca. −0.678 V vs. Ag|AgCl, has been reported [67,68].

When the potential of the working electrode is switched to +0.85 V vs. Ag|AgCl (Figure 2b, red line), electrooxidation of pyrrole to polypyrrole occurs as the main reaction. More detailed analysis of the current density recorded vs. time (Figure 2b, blue line) indicated that, at the beginning of the positive potential pulse, the current density increases, and a small decrease is then observed due to the steady-state growth of the polymer layer and a monomer loss at the electrode–electrolyte interface. Moreover, a side reaction, resulting in nickel hydroxide electro-conversion into NiOOH [69], might occur at the beginning of the positive potential pulse, when the electrolyte is still in contact with Ni(OH)_2_ formed previously.

**Figure 3:**
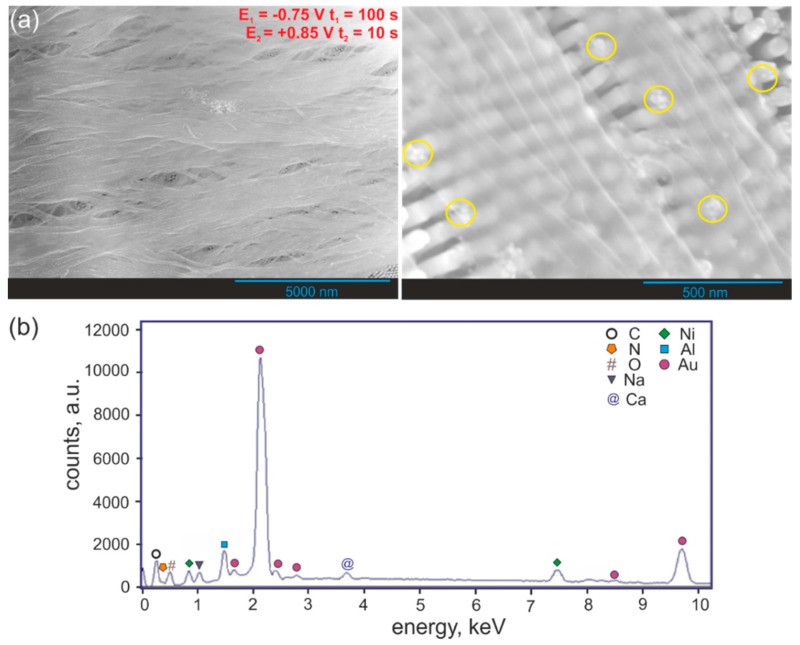
Low magnification (left-hand side) and high magnification (right-hand side) FE-SEM images of the PPy-Ni(OH)_2_ nanowire array (**a**) together with EDS analysis of hybrid nanowires (**b**). The presence of Ni(OH)_2_ nanoparticles is marked with yellow ellipses.

The morphology of hybrid nanowires fabricated via alternating cathodic reduction of Ni^2+^ and anodic polymerization of PPy is shown in Figure 3a. As one can see in Figure 3a, the obtained hybrid PPy-Ni(OH)_2_ nanowires have a uniform diameter and are smooth and homogeneous in shape. After closer inspection of the FE-SEM images (right-hand side image in Figure 3a), there are visible Ni(OH)_2_ nanoparticles inside the PPy matrix (marked with yellow circles).

The EDS analysis was performed (Figure 3b) to confirm the composition of hybrid PPy-Ni(OH)_2_ nanowires synthesized in the Watts-type solution containing 0.15 M Py + 0.10 M LiClO_2_ + 0.10 M Na_2_CO_3_. As expected, the resulting product of the synthesis is composed of carbon, nitrogen, oxygen, and nickel. Additionally, the EDS spectrum revealed the presence of Na, Al, Au, and Ca. The presence of sodium can be attributed to sodium hydroxide that was used for the AAO template removal. Incomplete removal of the AAO template is indicated by the presence of Al peak in the EDS spectrum. The peak assigned to Au, observed in the EDS spectrum, originates from the thin Au layer sputtered before electrodeposition. The peak assigned to calcium is, in fact, the sum peak of Si generated from the detector.

**Figure 4:**
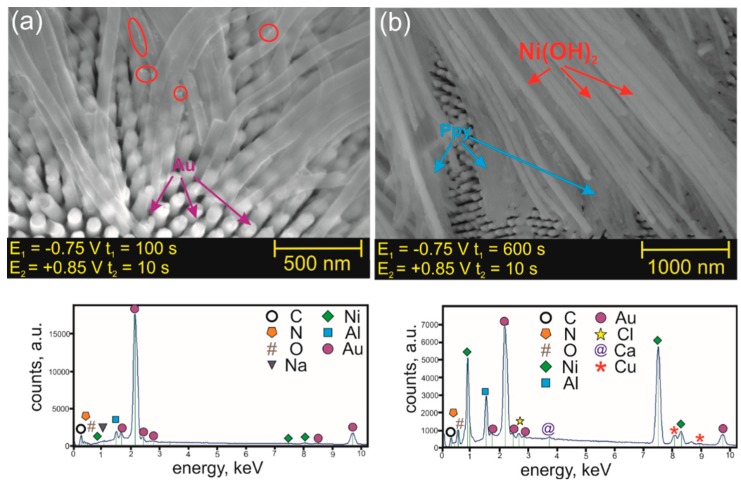
FE-SEM images (top images) together with their EDS analyses (bottom images) of hybrid PPy-Ni(OH)_2_ nanowires formed at different durations of the cathodic potential pulse. The duration of the cathodic pulse was (**a**) 100 s and (**b**) 600 s. The duration of the anodic pulse was 10 s for (a) and (b). The presence of Ni(OH)_2_ nanoparticles is marked with red ellipses.

Figure 4 shows the comparison of hybrid PPy-Ni(OH)_2_ nanowires obtained by pulse alternating electrodeposition with different durations of the cathodic pulse. As one can see from FE-SEM images, Ni(OH)_2_ segments are significantly longer (brighter, non-transparent nanowires in Figure 4b), while the polypyrrole segments are shorter and not well-distributed (darker, partially transparent nanowires in Figure 4b) when the time of the cathodic pulse was extended to 600 s.

Interestingly, for PPy-Ni(OH)_2_ nanowires deposited during the shorter cathodic pulse, i.e., 100 s (Figure 4a), no clear Ni(OH)_2_ segments are visible along the nanowire long axis. Ni(OH)_2_ nanoparticles (mark with red circles/ellipses) are observed only. Therefore, it is obvious that the shape and amount of deposited Ni(OH)_2_ material is strongly dependent on the duration of the cathodic pulse. The EDS spectra presented in Figure 4a,b proved that a higher amount of nickel (more intense Ni peaks) was deposited when the duration of the cathodic pulse was longer. A high-intensity oxygen peak observed in the EDS spectrum for the nanowires deposited with the cathodic pulse duration of 600 s confirmed that deposition of nickel hydroxide occurred. The presence of carbon and nitrogen in both EDS spectra (Figure 4) indicate the formation of polypyrrole independently of the cathodic pulse duration. It can therefore be concluded that, in order to obtain Ni(OH)_2_ nanoparticles dispersed in polypyrrole network, short cathodic pulses should be applied during electrodeposition.

**Figure 5:**
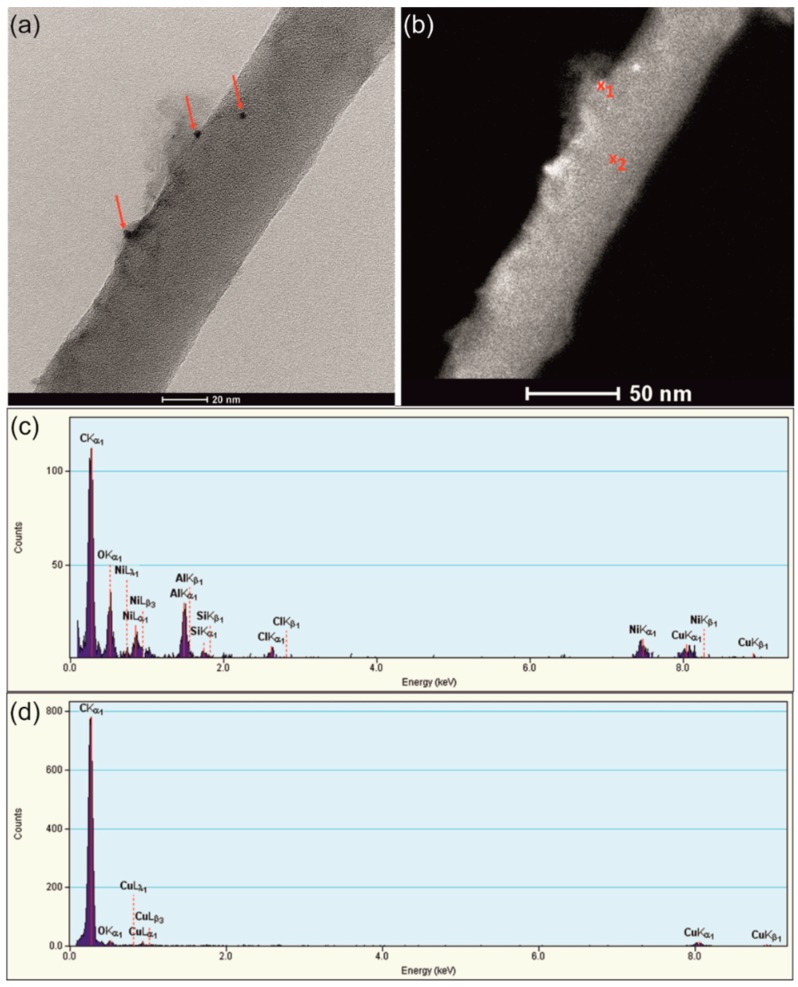
BFTEM image of the single hybrid PPy-Ni(OH)_2_ nanowire (**a**,**b**) together with EDS analyses of points marked in figure (b) as x_1_ (**c**) and x_2_ (**d**).

The dispersion of Ni(OH)_2_ nanoparticles inside the PPy matrix was studied using TEM as well (Figure 5).

Figure 5a presents a bright field transmission electron microscopy (BFTEM) image of the single PPy nanowire encrusted with Ni-rich nanoparticles (dark contrast features marked by red arrows). The size of the observed nanoparticles ranged from 2 to 7 nm. The EDS point analysis (taken from points marked in Figure 5b of the material) showed the presence of nickel and oxygen in the particles (Figure 5c) and the lack of impurities inside the PPy nanowire itself (Figure 5d). The copper signal present in EDS spectra for both nanoparticles and nanowires comes from Cu grids, used as material support for TEM observations. Incomplete removal of the AAO template is indicated by the presence of Al peak in the EDS spectrum.

**Figure 6:**
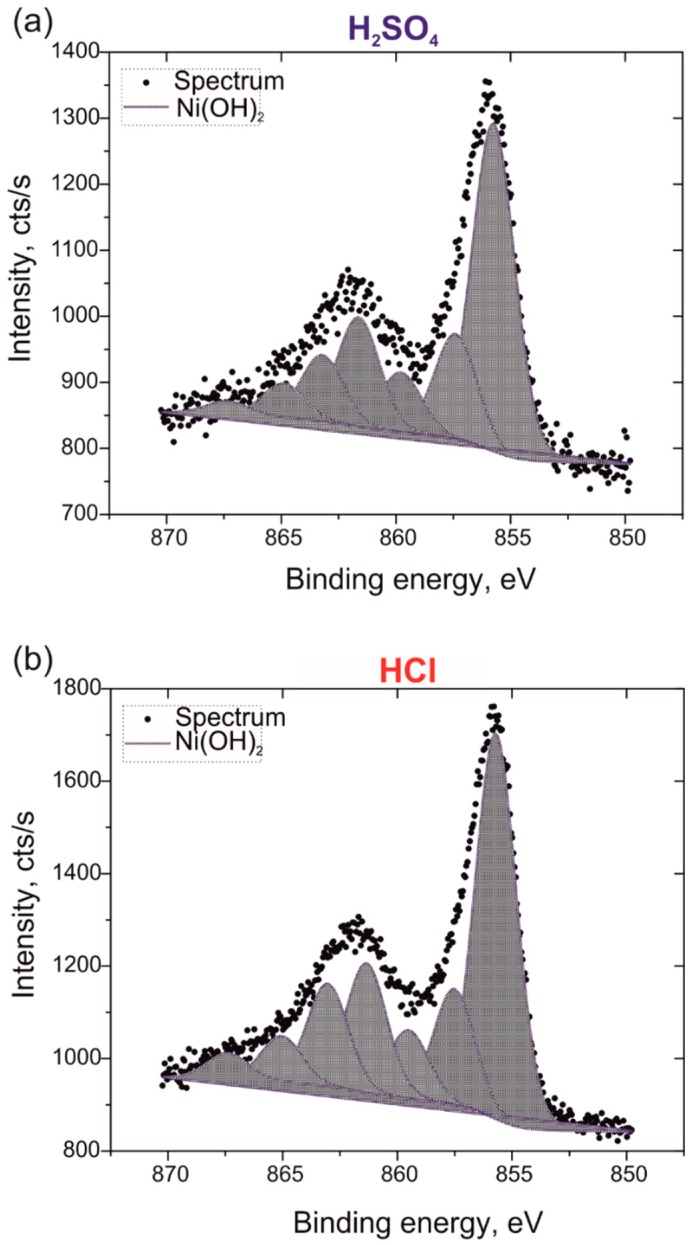
High-resolution deconvoluted XPS spectra of Ni 2p_3/2_ for hybrid PPy-Ni(OH)_2_ nanowires deposited in the presence of H_2_SO_4_ (**a**) and HCl (**b**) in the Watts-type electrolyte containing 0.15 M Py + 0.10 M LiClO_2_ + 0.10 M Na_2_CO_3_.

X-ray photoelectron spectroscopy (XPS) analyses were performed to confirm the presence of nickel hydroxide phase in electrodeposited nanowires. Figure 6 shows high-resolution XPS spectra of the Ni 2p_3/2_ region along with the deconvoluted peaks for hybrid PPy-Ni(OH)_2_ nanowires deposited with the cathodic pulse duration of 100 s from the Watts-type electrolyte acidified with H_2_SO_4_ and HCl. Inspection of peak positions indicates that the predominant nickel form on the surface of both samples is nickel hydroxide (main line centered at ~855 eV) [70], but the presence of low amounts of γ-NiOOH and/or β-NiOOH nickel hydroxides cannot be excluded [71]. However, due to the very weak signal from these species and multiplet splitting phenomenon for Ni 2p_3/2_ line, the unique attribution of peaks to these states is indistinguishable from Ni(OH)_2_. The overall atomic concentration of nickel species at the surface equals to 6.6% and 12.6% for PPy-Ni(OH)_2_ nanowires deposited in the presence of H_2_SO_4_ (Figure 6a) and HCl (Figure 6b), respectively.

**Figure 7:**
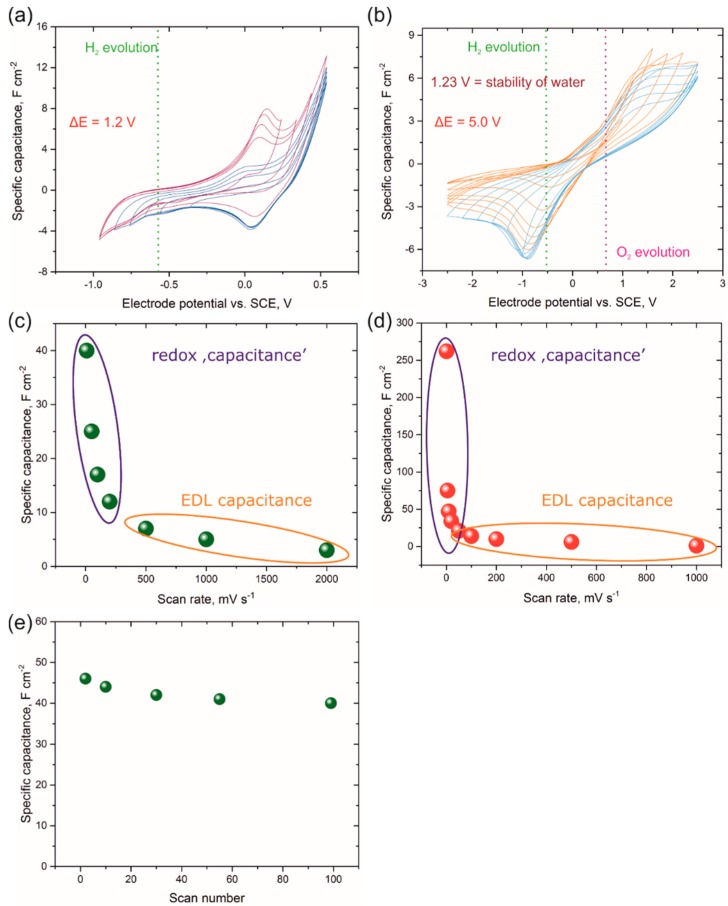
Three-electrode cyclic voltammetry scans (5 mV s^−1^) on the PPy (**a**) and hybrid PPy-Ni(OH)_2_ (**b**) nanowire array electrodes in a 1 M Li_2_SO_4_ + 0.19 M 1,4-dihydroxybenzene aqueous solution with a gradual shift to negative and positive potential. A real capacitance as a function of scan rate for the PPy (**c**) and PPy-Ni(OH)_2_ (**d**) nanowire array electrodes. The specific capacitance of the PPy nanowire array electrode vs. scan number dependence (**e**).

The electrochemical potential window for the PPy (Figure 7a) and PPy-Ni(OH)_2_ (Figure 7b) nanowire array electrodes were examined by cyclic voltammetry measurements carried out in a 1 M Li_2_SO_4_ + 0.19 M 1,4-dihydroxybenzene aqueous solution. It was decided that cyclic voltammetry will be the most appropriate method for fundamental evaluation of the material since all of the expected processes are potential-controlled. The specific capacitance of obtained electrodes was calculated using the following formula:

(4) $$C=\frac{i}{P_{el}\cdot \nu }$$

where *C* (F cm^–2^) is the specific capacitance, *i* (A) is the current, *P_el_* (cm^2^) is the surface area of the electrode, and *ν* (V s^−1^) is the scan rate.

Application of the electrolyte containing redox-active specimen was done in order to ensure the protons for polymer doping-dedoping; one should be aware that, in the pH-neutral electrolyte, the pH might become alkaline during hydrogen evolution and the polymer might become inactive (non-conductive). A quinone–hydroquinone redox couple has already been applied as an electrolyte additive for electrochemical capacitors or as a specimen grafted on the electrode surface [72,73,74,75,76,77]. It was demonstrated that this redox couple contributes to the total capacity of the system with a faradaic type of charge storage.

The comparison of Figure 7a,b shows that the hybrid PPy-Ni(OH)_2_ nanowire array electrode has a wider potential window (ΔE = 5.0 V in three-electrode system); definitely, the high value of the potential window originates from the high overpotentials of the oxygen or hydrogen evolution on the nanostructured surface and three-electrode experiment configuration (with excess of the electrolyte). For the PPy nanowire array electrode, the stability window did not exceed 1.2 V. Although the potential window for PPy nanowire array electrode is in good agreement with the literature data [78,79,80], it is still insufficient for further applications, especially due to the fact that capacitance properties of the PPy electrode are usually tested in acidic media [78,80].

When the modified electrode, i.e. the hybrid PPy-Ni(OH)_2_ nanowire array, was tested in the same system, the potential window was extended more than twice. The theoretical thermodynamic stability of water, i.e., 1.23 V, determines the maximum voltage limit to less than 1 V; however, by modifying electrolyte or electrode composition [81], the potential window can be extended. In the case of the PPy-Ni(OH)_2_ electrode, the reason for such a wide potential window is the pseudocapacitive behavior of Ni(OH)_2_. It is assumed that the redox reaction of Ni(OH)_2_ precedes the oxygen evolution and protects the electrode against overcharging. The main reaction responsible for pseudocapacitance of the PPy-Ni(OH)_2_ nanowire array electrode is as follows (Equation (5)):

(5) $$Ni{\left(OH\right)}_{2}+OH^{-}↔NiOOH+H_{2}O+e^{-}.$$

For both electrodes, cyclic voltammetry experiments at selected scan rates were conducted in a 1 M Li_2_SO_4_ + 0.19 M 1,4-dihydroxybenzene aqueous solution. The specific capacitance, calculated using cyclic voltammetry records was 25 F cm^−2^ for PPy and 75 F cm^−2^ for the PPy-Ni(OH)_2_ nanowire array electrode at a scan rate of 20 mV s^−1^. The dependence of specific capacitance vs. scan rate (Figure 7c,d) reveals that the main capacitance (more precisely, capacity) in both systems originates from the redox reaction, while EDL capacitance has a small impact for the overall value of capacitance. However, for the hybrid PPy-Ni(OH)_2_ nanowire array electrode, the maximum capacitance value observed for the lowest scan rate was more than five times higher than that of the PPy nanowire electrode. The cut-off value for the EDL capacitance was established in the region where the capacitance values are independent of the scan rate applied.

Cyclic voltammetry explored the cyclic stability of the PPy nanowire array electrode in a 1 M Li_2_SO_4_ + 0.19 M 1,4-dihydroxybenzene aqueous solution in the three-electrode system. Figure 7e shows the cyclic stability of the PPy nanowire array electrode up to 100 cycles. After 100 voltammetry cycles, a capacitance fade of about 13% was observed.

## 4. Conclusions

Successful fabrication of new material, hybrid PPy-Ni(OH)_2_ nanowires, inside the AAO template through one-step pulse electrodeposition of Ni(OH)_2_ and PPy from a single bath solution is presented. The shape and amount of deposited Ni(OH)_2_ material depend strongly on the duration of the cathodic pulse. Therefore, in order to obtain Ni(OH)_2_ nanoparticles dispersed in a polypyrrole network, short cathodic pulses should be applied during electrodepositions. The morphology of hybrid PPy-Ni(OH)_2_ nanowires was characterized by SEM and TEM analyses, which confirmed the existence of metal hydroxide nanoparticles dispersed inside the PPy matrix. EDS confirmed the chemical composition of obtained hybrid nanowires. The existence of carbon and nitrogen in the EDS spectrum of synthesized PPy-Ni(OH)_2_ hybrid nanowires confirmed the presence of PPy, while nickel and oxygen proved the presence of Ni(OH)_2_. XPS analyses supported these data. The fabricated PPy-Ni(OH)_2_ nanowire array electrode shows a broad available potential window and a higher pseudocapacitance in comparison with the non-modified PPy nanowire array electrode. The available potential window for the PPy-Ni(OH)_2_ nanowire array electrode is 5 V in the three-electrode system while for the non-modified PPy electrode is 1.2 V. The capacitance value, calculated from cyclic voltammetry curves at 20 mV s^−1^, was 25 F cm^−2^ for PPy and 75 F cm^−2^ for PPy-Ni(OH)_2_ array electrodes. The enhanced-capacitance behavior of the hybrid PPy-Ni(OH)_2_ nanowire array electrode is a consequence of the existence of nickel hydroxide nanoparticles. We have successfully synthesized a hybrid PPy-Ni(OH)_2_ nanowire array and proven that it is a promising material for energy storage applications.
